# Summary results of the 2014-2015 DARPA Chikungunya challenge

**DOI:** 10.1186/s12879-018-3124-7

**Published:** 2018-05-30

**Authors:** Sara Y. Del Valle, Benjamin H. McMahon, Jason Asher, Richard Hatchett, Joceline C. Lega, Heidi E. Brown, Mark E. Leany, Yannis Pantazis, David J. Roberts, Sean Moore, A Townsend Peterson, Luis E. Escobar, Huijie Qiao, Nicholas W. Hengartner, Harshini Mukundan

**Affiliations:** 1 Analytics, Intelligence, and Technology Division, Los Alamos National Laboratory, P.O. Box 1663, Bikini Atoll Road, Los Alamos, New Mexico, 87544 USA; 2 Theoretical Division, Los Alamos National Laboratory, P.O. Box 1663, Bikini Atoll Road, Los Alamos, New Mexico, 87544 USA; 3 Leidos Supporting Biomedical Advanced Research and Development Authority, 200 Independence Avenue, S.W., Washington, District of Columbia, 20201 USA; 4 Office of the Assistant Secretary for Preparedness and Response, U.S. Department of Health and Human Services, 200 Independence Avenue, S.W., Washington, District of Columbia, 20201 USA; 5 Department of Mathematics, University of Arizona, 617 N. Santa Rita Ave, Tucson, Arizona, 85721 USA; 6 Epidemiology and Biostatistics Department, University of Arizona, 1295 N. Martin Ave, Tucson, Arizona, 85724 USA; 7 Utah Valley University, 800 W University Pkwy, Orem, Utah, 84058 USA; 8 Department of Mathematics and Statistics, University of Massachusetts, 710 N. Pleasant St, Amherst, Massachusetts, 01003 USA; 9 Present Address: Institute of Applied and Computational Mathematics, Foundation for Research and Technology - Hellas, Heraklion, Greece; 10 NHS Blood and Transplant-Oxford, BRC Haematology Theme and Radcliffe Department of Medicine, John Radcliffe Hospital, Headley Way, Oxford, OX3 9BQ UK; 11 Department of Biological Sciences, University of Notre Dame, Notre Dame, 46556 IN USA; 12 Biodiversity Institute, University of Kansas, 1345 Jayhawk Blvd, Lawrence, Kansas, 66045 USA; 13 Department of Fish and Wildlife Conservation, Virginia Tech, Blacksburg, 24061 VA USA; 14 Institute of Zoology, Chinese Academy of Sciences, 1 Beichen West Road, Chaoyang District, Beijing, 100101 China; 15 Chemistry Division, Los Alamos National Laboratory, P.O. Box 1663, Bikini Atoll Road, Los Alamos, New Mexico, 87544 USA

**Keywords:** Chikungunya, Forecasting, Morphological models, Mechanistic models

## Abstract

**Background**: Emerging pathogens such as Zika, chikungunya, Ebola, and dengue viruses are serious threats to national and global health security. Accurate forecasts of emerging epidemics and their severity are critical to minimizing subsequent mortality, morbidity, and economic loss. The recent introduction of chikungunya and Zika virus to the Americas underscores the need for better methods for disease surveillance and forecasting.

**Methods**: To explore the suitability of current approaches to forecasting emerging diseases, the Defense Advanced Research Projects Agency (DARPA) launched the 2014–2015 DARPA Chikungunya Challenge to forecast the number of cases and spread of chikungunya disease in the Americas. Challenge participants (*n*=38 during final evaluation) provided predictions of chikungunya epidemics across the Americas for a six-month period, from September 1, 2014 to February 16, 2015, to be evaluated by comparison with incidence data reported to the Pan American Health Organization (PAHO). This manuscript presents an overview of the challenge and a summary of the approaches used by the winners.

**Results**: Participant submissions were evaluated by a team of non-competing government subject matter experts based on numerical accuracy and methodology. Although this manuscript does not include in-depth analyses of the results, cursory analyses suggest that simpler models appear to outperform more complex approaches that included, for example, demographic information and transportation dynamics, due to the reporting biases, which can be implicitly captured in statistical models. Mosquito-dynamics, population specific information, and dengue-specific information correlated best with prediction accuracy.

**Conclusion**: We conclude that with careful consideration and understanding of the relative advantages and disadvantages of particular methods, implementation of an effective prediction system is feasible. However, there is a need to improve the quality of the data in order to more accurately predict the course of epidemics.

## Background

Mathematical models for infectious diseases have been used to gain insight into disease dynamics for more than a century [1–4]. However, only recently have models and systems begun to be designed specifically for the task of providing regularly updated quantitative forecasts of infectious disease spread that are analogous to those available for weather prediction. Forecasting approaches vary substantially in both method and complexity; for example, some use human judgment or prediction markets, some use purely statistical or machine learning approaches, and others rely upon disease transmission models of varying complexity [5–8].

In parallel, recent experiences responding to outbreaks have highlighted the significant utility of infectious disease forecasts to support decision-making [9, 10]. Models provide critical insight in the face of limited data by forecasting the international spread of viruses, illustrating the value of different mitigation strategies, and assessing the risk of continued danger in cases such as the 2009 influenza pandemic [11, 12]. Early predictions for the 2014-2015 Ebola outbreak in West Africa indicated that incidence would continue to grow rapidly unless significant mitigation measures were undertaken [13]. This information helped galvanize the international response to the crisis and indicate the importance of rapid deployment of resources. As the outbreak progressed, incidence forecasts were used to inform the planning and execution of clinical trials for vaccines and therapeutics by ensuring that activities were responding to the rapidly changing situation and that decision makers had adequate time to develop contingency plans [14, 15].

Disease forecasting has received significant attention among the mathematical epidemiological community as well as decision makers. For example, the 2012 National Strategy for Biosurveillance [16] specifically identified forecasting as one of the core functions of a national biosurveillance enterprise. Building upon this, the 2013 National Biosurveillance Science and Technology Roadmap identified several key research priorities, including additional research and development for disease forecasting technology, which are critical to achieving the overall goal of providing decision makers with more accurate and timely information during biological incidents.

In response to this madate, several United States (US) Government agencies have conducted challenge and prize competitions that involved infectious disease forecasting in an effort to help mature operational forecasting technologies. The Center for Disease Control and Prevention has organized consecutive challenges for the 2013-2018 influenza seasons that have focused on predicting the timing and intensity of influenza-like illness (ILI) in the US at the regional level [17, 18]. In 2015, several departments in the US Government joined together with the support of the National Science and Technology Council to launch an open dengue challenge that strove to forecast disease incidence using previously unpublished data from Peru and Puerto Rico [19]. The 2014-2015 DARPA Chikungunya Challenge was conceived as an effort to mobilize a wide variety of participants to foster innovation and advance the state of the art by attempting to predict chikungunya incidence across the Americas [20].

Nonetheless, significant challenges remain for the development of operational forecasting as a mature technology [21]. The fundamental science of forecasting needs to be developed and supported by a robust research program. Data availability is often limited, especially during outbreak responses, and this hampers the ability to provide critical insights in a timely fashion. While some decision makers have embraced the use of modeling and forecasting, others remain skeptical, having been presented with forecasts that were inaccurate and that did not make the inherent underlying uncertainties clear.

This manuscript summarizes the challenge and provides a description of the top six solver submissions including data sources and methodologies.

### Chikungunya challenge

Chikungunya is a mosquito-borne viral infection of humans. Although rarely fatal, chikungunya is an emerging, debilitating viral disease that is transmitted among humans by mosquitoes [22]. There is no specific treatment for the disease, although palliative care has been shown to reduce its severity and duration. The chikungunya virus (CHIKV) was originally detected in Tanzania in 1952, with the name meaning ‘to become contorted’ in the Kimakonde language of Mozambique, referring to the effects of severe joint pain [23]. Chikungunya expanded to Asia and the Indo-Pacific islands, causing notably large outbreaks over the past 10-20 years.

The CHIKV epidemic was well suited for this Challenge because its spread to the Western Hemisphere had been expected for some years and presented a valuable opportunity to evaluate disease progression in a naive population. Further, there was a pre-existing reporting system via the Pan American Health Organization (PAHO) in place for tracking disease incidence across the Americas. The goal of the DARPA Chikungunya Challenge was to evaluate state-of-the-art epidemic modeling methods to forecast outbreaks of CHIKV throughout the Americas, to compare modeling strategies, and to provide insight into how different data streams could be incorporated into these models. The Challenge provided a baseline of current forecasting capabilities for infectious diseases and their applicability for vector-borne infectious diseases.

### Design and execution of the DARPA Chikungunya challenge

The introduction of CHIKV into the Western Hemisphere had been anticipated, and the first case was recorded in Saint Martin in December 2013 [24]. Its emergence in the Caribbean caused substantial morbidity in the population and concern about subsequent spread in the Americas. After the first cases were reported in December 2013, the virus spread throughout the Eastern Caribbean islands and into Central and South America, reaching the United States in mid-July, 2014. Since then, Zika has been detected in several countries and territories of the Americas [25]. As of epidemiological week 35 of 2014 (September 18, 2014), when the DARPA Chikungunya Challenge was initiated, 659,367 cases, including 37 deaths, had been reported in the Americas. The disease was determined to be an ideal candidate for the DARPA Chikungunya Challenge because of the predictable spread of the virus among an immunologically naive population, and the availability of incidence data reported by participating countries to PAHO [25].

The Department of Defense’s (DOD) role in global health includes conducting timely, relevant, and comprehensive health surveillance to promote, maintain, and enhance the health of both the military and associated populations. Tracking disease outbreaks and emergence of new pathogens is an intrinsic component of this effort. Force health protection and readiness, protection of civilian populations, medical stability operations, and partnership engagement are key components to this mandate. Conducting health surveillance that can detect, contain, and prevent impacts of intentional or natural biological events is a critical part of the DOD’s ability to maintain force health while promoting stability and security abroad. To accomplish this, there needs to be a proactive approach to anticipating the geographic and temporal trajectory of infectious disease outbreaks.

Mathematical and statistical models (grouped under the morphological category in this manuscript) are used not only to forecast the spatial-temporal evolution of real world outbreaks, but also to estimate the potential value of mitigation efforts. The latter requires an accurate understanding of both public policy and the behavior of people in novel situations. A further challenge is how existing methods account for delayed reporting and underreporting, and how to use additional data streams to reduce systematic errors (bias) and forecasting uncertainties. The DARPA Chikungunya Challenge addressed this data gap by promoting innovation in data integration techniques.

**Fig. 1:**
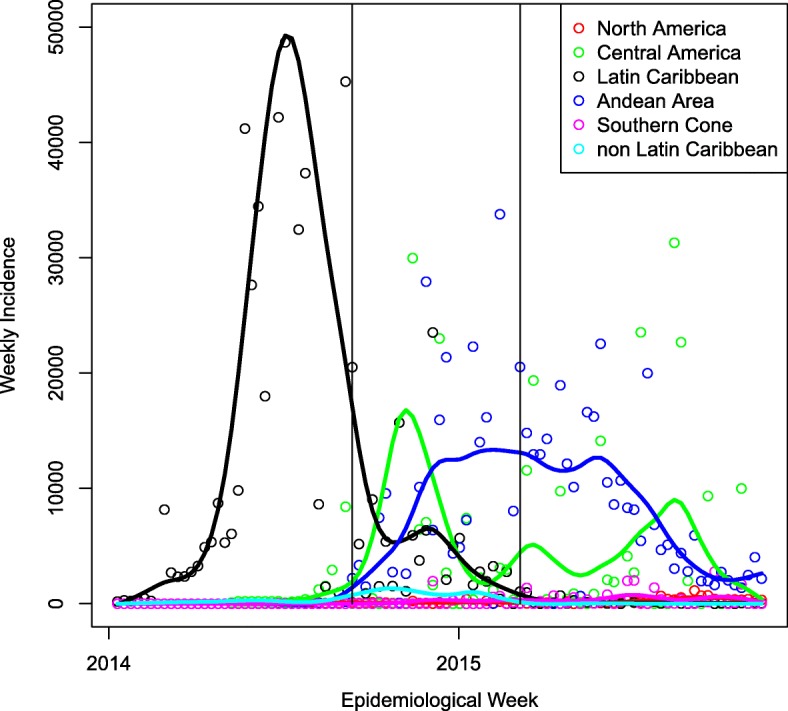
Weekly incidence of chikungunya cases, aggregated by region from PAHO reports (symbols) and smoothed epidemic curves (lines). The two vertical lines show the beginning and end of the prediction period for the DARPA Challenge

The DARPA Chikungunya Challenge asked participants to forecast the cumulative total cases (suspected and confirmed, the latter including imported-confirmed) per week per country. A format was selected to inspire innovative approaches and encourage non-traditional participants, forecasting approaches, and data sources to improve overall infectious disease forecasting capabilities. The forecast submissions were evaluated and scored on a weighted basis (Table 1). The forecasts were submitted at various stages of the epidemic progression across the Americas (Fig. 1). The figure provides information on the epidemic progression as PAHO reports during the time of the reporting [26]. Evaluation of methodology was performed by a panel of non-competing government subject matter experts in infectious disease modeling, CHIKV, and other vector-borne diseases.

**Table 1:** Description of the DARPA Chikungunya Challenge deliverables and points description

Deliverable	Due date	Content	Max
			Points
1	September 1, 2014	Initial methodology, documentation, and data sources	5
2	Septebmer 8, 2014	Forecast for 6-month period (Epidemic week 36-9)	5
3	October 1, 2014	Forecast for peak new cases	10
4	October 1, 2014	Forecast for 5-month period (Epidemic week 36-9)	15
5	November 1, 2014	Forecast for 4-month period (Epidemic week 36-9)	20
6	December 1, 2014	Forecast for 3-month period (Epidemic week 36-9)	15
7	January 1, 2015	Forecast for 2-month period (Epidemic week 36-9)	10
8	February 1, 2015	Forecast for 1-month period (Epidemic week 36-9)	5
9	February 1, 2015	Final methodology, documentation, and data sources	15
		Maximum total points	100

Accuracy was scored based on the predicted number of cases and spread of CHIKV in the Americas compared to weekly publicly-available PAHO reporting of suspected and confirmed cases. Participants were encouraged to utilize any publicly available data for modeling and forecasting such as climate, clinical surveillance data, genetic information, and social media. Proprietary data were permitted for incorporation into models if obtained independently by participants. Participants were not required to disclose the content of proprietary data but had to include a detailed description of how it was obtained and used in the Challenge methodology deliverables. The methodology reports required sections describing: (1) data sources used, (2) model robustness, (3) applicability, (4) presentation, and (5) computational requirements.

## Methods

### Summaries of participants’ approaches

DARPA awarded cash prizes to six leading participants, including $150,000 for first place, $100,000 for second place, and $50,000 to each of four honorable mentions. The leading participants used varying methodologies and model types to inform their forecasts. The following are descriptions of their overall approach, methodologies to forecast the spread of chikungunya in the Americas, and a brief summary of their results.

### First place submission (henceforth participant 1)

*A simple model for the recent outbreaks of chikungunya in the Americas*

*Modeling Approach*: Participant 1 relied on estimating the growth rate *G*(*N*) of the outbreak in each country as a function of *N*, where *G*=*dN*/*dt*,*N* is a smooth interpolation of the total number of cases reported on the PAHO website, and *t* is time in weeks. The function *G* implicitly reflects the combined effects of the meteorological, geographic, human, and vector characteristics that describe vector borne diseases. Participant 1 fitted *G* to a quadratic or piecewise quadratic function *G*_*f*_, which describes *N* as proportional to the number of infected and recovered individuals in an Susceptible-Infectious-Recovered (SIR) model [27]. Participant 1 solved the differential equation *dN*/*dt*=*G*_*f*_(*N*) and chose parameters in the expression of *G*_*f*_ as to optimize both (*C*_1_) (i.e., how well *G*_*f*_(*N*) approximates G(N)) and (*C*_2_) (i.e., how well *N*(*t*), obtained from solving *dN*/*dt*=*G*_*f*_(*N*), fits the reported cumulative epidemiological curve) [28].

**Fig. 2:**
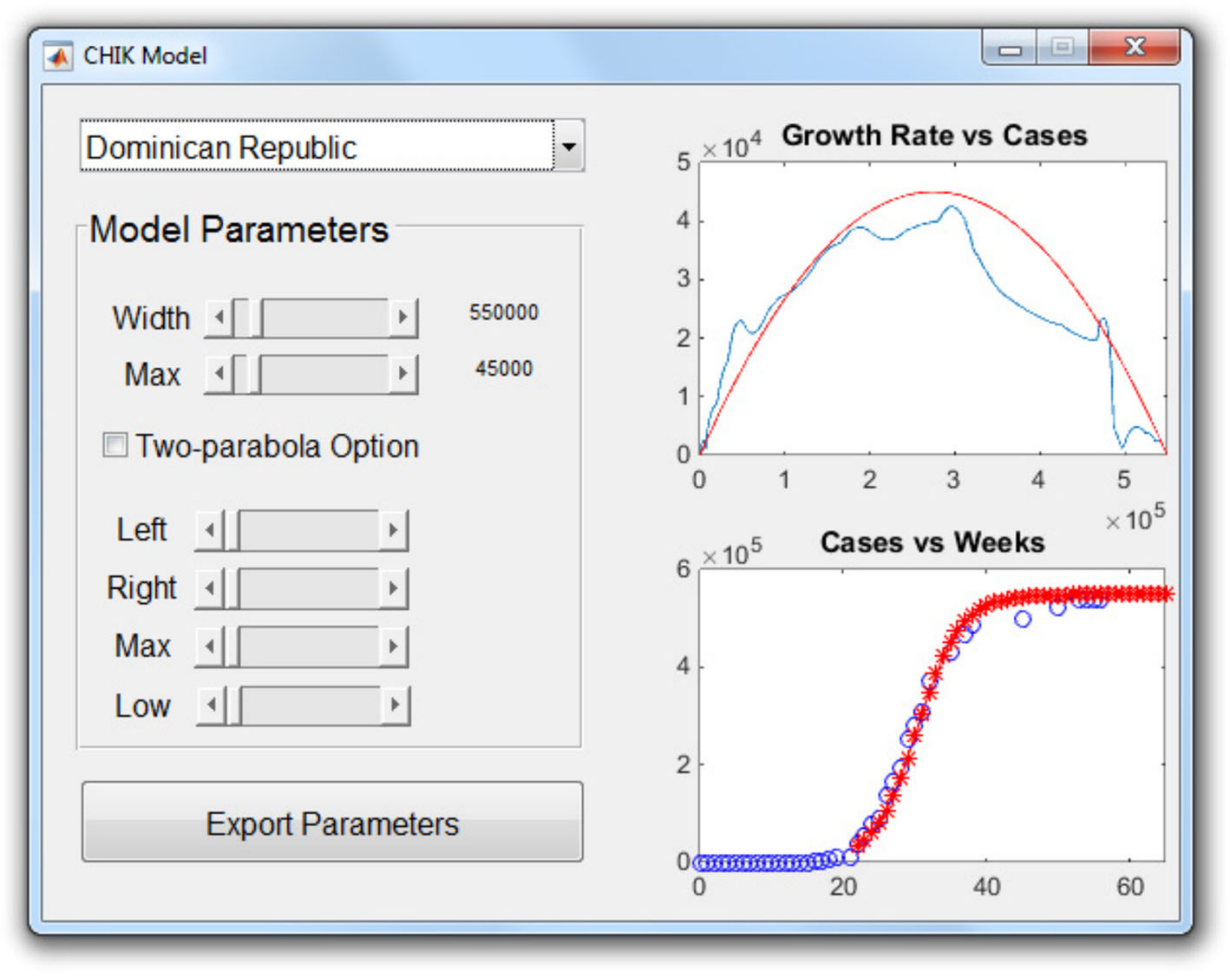
The MATLAB interface used for the model (developed by Participant 1).The growth rate *G*(*N*) for the Dominican Republic is shown by the solid blue curve. The inverted parabola in red represents its quadratic approximation *G*_*f*_(*N*). Parameter values are set by the sliders on the left. The bottom right plot compares the predicted and observed cumulative epidemiological curves: the red stars are the model predictions obtained by solving *dN*/*dt*=*G*_*f*_(*N*); the reported data (from PAHO) are shown as blue circles

*Results*: Model parameters were estimated by hand, with the help of a MATLAB graphical user interface, displayed in Fig. 2. The top right plot shows how *G*(*N*) (blue solid curve) for the Dominican Republic may be approximated by a quadratic function (inverted parabola in red). Parameter values are set by the sliders on the left. The bottom right plot compares the predicted and observed cumulative epidemiological curves: the red stars are the model predictions obtained by solving *dN*/*dt*=*G*_*f*_(*N*); the reported data are shown as blue circles. By observing how changes in the model parameters affected these plots, parameter values that best fitted the data for each country were selected. Participant 1 organized the PAHO countries into groups, depending on dengue and CHIKV incidence and on whether a quadratic or piecewise quadratic fit for *G* was used. Attempts to connect these groups to economic (Gini Coefficient, per capita Gross Domestic Product), demographic (population density and percent of population living in urban areas), connectivity (number of ports, number of port calls, and distance between islands), and health indices (infant mortality and life expectancy) were unsuccessful.

### Second place submission (henceforth participant 2)

*Predicting the spread of chikungunya using a logistic S-curve*

**Fig. 3:**
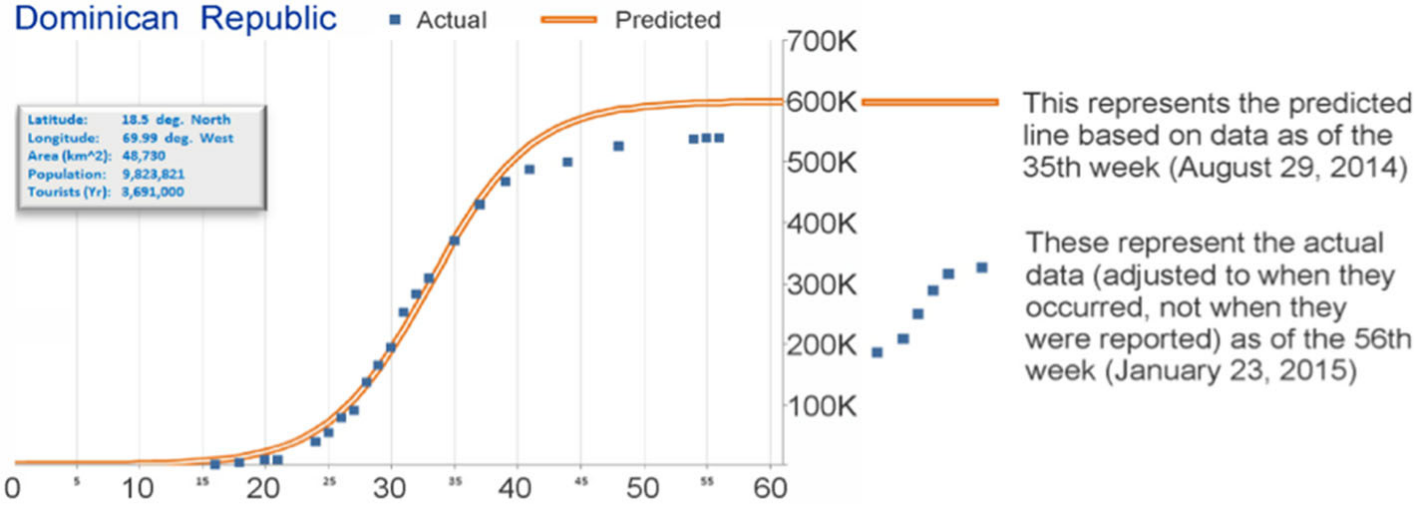
The straight orange line represents the predicted line based on data as of the 35th week (August 29th, 2014), by participant 2. The blue squares represent the actual data (adjusted to when they occurred, not when they were reported) as of the 56th week (January 23rd, 2015)

*Modeling Approach*: Participant 2 used a Bounded Geometric Growth approach (shown by a logistic function or S-curve on Fig. 3) to model CHIKV across the americas. Participant 2 used a macro-enabled Excel workbook to manually fit each curve to the PAHO data for each country.

*Results*: This approach described the overall dynamics for about half the countries. The results show that the model worked best for countries with higher incidence than for countries with low incidence.

### Honorable mention #1 (henceforth participant 3)

*Forecasting chikungunya fever*

**Fig. 4:**
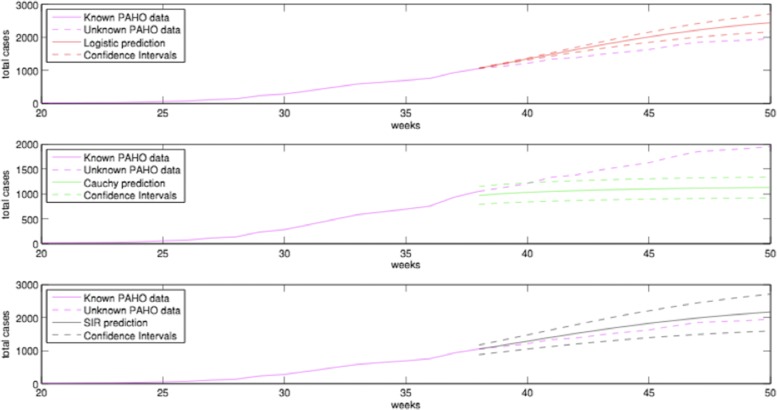
Smoothed PAHO data (magenta) and the logistic model (red), Cauchy model (green) and SIR model (black) for the USA’s total cases from Participant 3. Data from the first 38 weeks were used for the prediction. The upper and lower bounds were computed from the covariance matrix of the estimated parameter vector

*Modeling Approach*: Participant 3 implemented three different predictive models for each country, namely the logistic model, the Cauchy model, and an epidemiological SIR model, which were fitted to the smoothed PAHO data. The basic assumption that all predictive models have is that the total cases for each country is a sigmoidal function of time (Fig. 4). The parameters of each model were estimated by regularized weighted non-linear least squares. In detail, the iterative Gauss-Newton algorithm was utilized for the minimization of the error (or cost) function. The weighting procedure assigns more weight to the recent data rather than to the past, modeling the fact that data from the far past contain less information about the future. Furthermore, due to the typical lack of enough data, especially at the early stages of an outbreak, the problem of minimization can be ill determined; therefore, the problem is regularized using Tikhonov (or ridge) regularization [29]. All considered, models had only three parameters to be estimated.

*Results*: The forecasts were obtained for each country by projecting the estimated predictive model to the future. Confidence intervals were provided for the estimated parameter vector based on the covariance matrix. The computed confidence intervals were able to create upper and lower bonds for the predicted values. Figure 4 shows the three-month forecasts for the USA. Notice that the SIR prediction has the best performance for the USA, but the logistic or Cauchy predictions were found to perform better in other countries.

### Honorable mention #2 (henceforth participant 4)

*A simple empirical approach to predict the spread of epidemics*

**Fig. 5:**
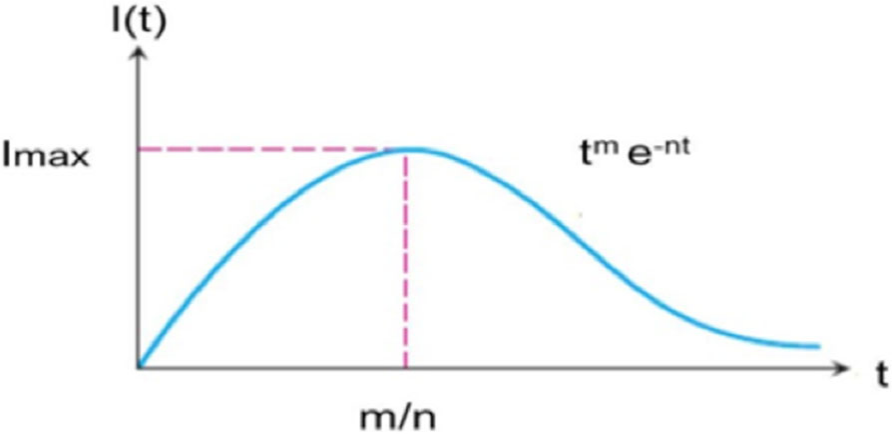
Empirical model for disease progression used by Participant 4. Incidence (*I*) is plotted as a function of time *I*(*t*), and can be fitted, *I*(*t*)=*At*^*m*^*e*^−*nt*^ where *A*, *m* and *n* are constants and *m*>0

*Modeling Approach*: Participant 4 used an empirical approach to fit the observed incidence provided by PAHO using the least-means squares. For epidemics where there is active transmission in a population, the incidence as a function of time *I*(*t*) can be fitted to incidence, *I*(*t*)=*At*^*m*^*e*^−*nt*^,where *A*, *m* and *n* are constants and *m*>0,as depicted in Fig. 5. The cumulative incidence for autochthonous and imported cases for each territory was obtained from the weekly PAHO data and used to derive the weekly incidence for each territory [30]. For simplicity, countries were considered to have either autochthonous transmission or imported cases. The cumulative number of cases was fitted to the incidence function for the model using the weekly incidence data derived from PAHO. Conditions were imposed to allow a solution to be derived. The solutions were found to be optimal when the total cases, and the cases in the last six weeks in predictions from the model were matched with observed data, and transmission was assumed to last no longer than one year [31]. Imported cases were predicted to follow the total infections in the region, and were scaled to the historical proportion of imported cases to total cases for each country.

*Results*: This simple and robust method provides satisfactory solutions, which may circumvent some of the problems of classical analytic methods for basic epidemics. The method outlined gives a good approximation for short-term forecasting especially with limited data but cannot give probabilistic forecasts nor provide an analytical model that can be refined using more detailed data of transmission, incident cases, and population movement.

### Honorable mention #3 (henceforth participant 5)

*Forecasting the Spread of Chikungunya Virus using a Coupled SEIR Transmission Model*

*Modeling Approach*: Participant 5 used a stochastic, mechanistic model of transmission dynamics in each locality to forecast chikungunya epidemics for each country and territory in the PAHO data. A susceptible-exposed-infectious-recovered (SEIR) transmission model was developed to describe viral transmission between human and mosquito populations [32]. People in the susceptible class experience a force of infection and become infected at a rate, $\lambda H = \alpha \beta _{1} Z^{\varphi _{1}} / N + \xi \phantom {\dot {i}\!}$ which depends on the biting rate of mosquitoes (*α*), the transmission efficiency of the virus from mosquito to humans (*β*_1_), and the number of infectious mosquitoes per human (*Z*/*N*). The force of infection scales non-linearly with the number of infectious mosquitoes ($\phantom {\dot {i}\!}Z^{\varphi _{1}}$), where *φ*_1_<1. The human force of infection also includes exposed individuals coming into the population from elsewhere at rate *ξ*, which was represented using a gravity model, with the rate entering the population from another locality dependent on the sizes of each population and inversely proportional to the distance between the two populations [33]. This mechanistic model was implemented in a state-space modeling framework with an imperfect observation process on top of the transmission dynamics and stochasticity in both the infection and observation processes. Model parameter values were estimated and then used to generate weekly forecasts using an iterated filtering method for calculating maximum likelihood estimates implemented in the pomp package in R [34].

**Fig. 6:**
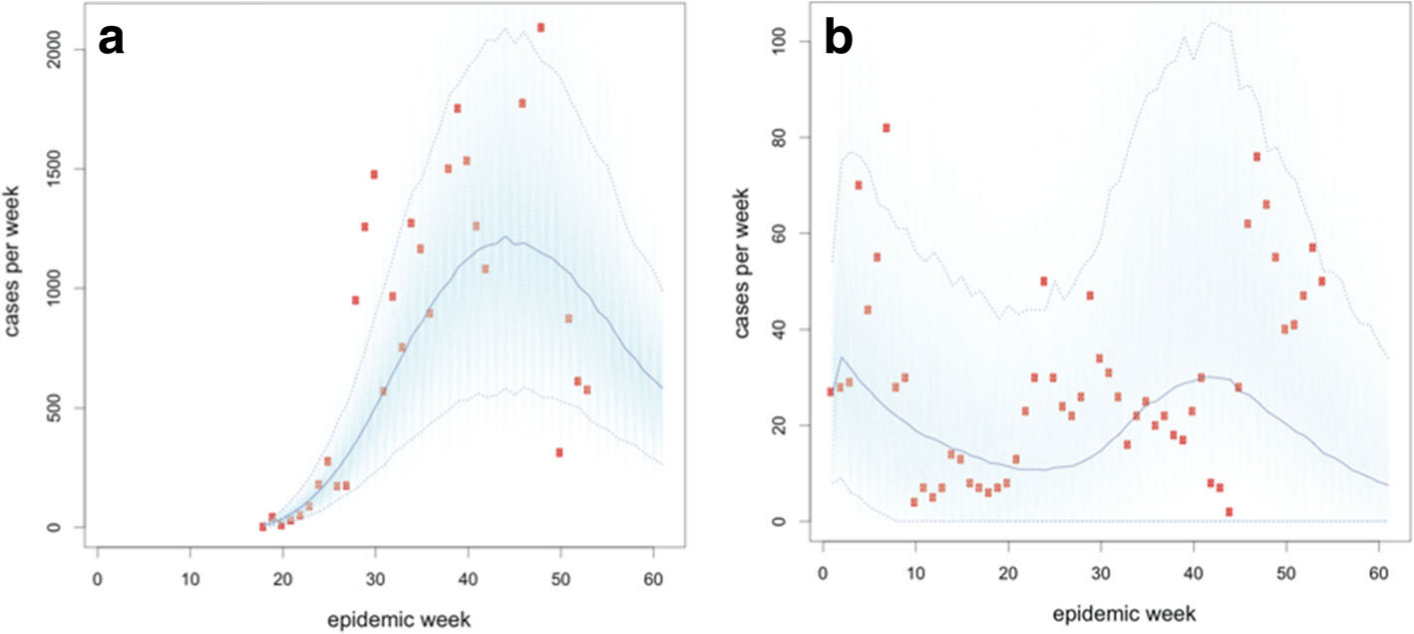
Weekly simulation of reported chikungunya cases in (**a**) Puerto Rico and (**b**) Saint Barthelemy from Participant 5. Simulations are one-month forecasts for February 2015. Red circles represent reported cases and each light blue line represents one of 2000 simulated outbreaks. Dark blue line is median used for prediction and dashed lines are 95% prediction intervals

*Results*: The weekly forecasts were calculated as the median of 2000 simulations (Fig. 6). The overall number of cases predicted was fairly accurate, particularly for the one to four month forecasts. The number of country forecasts that were significantly over or underestimated also decreased over time. In addition, five large outbreaks (> 1000 reported cases) were severely (> 50*%*) underestimated in the five-month forecast.

### Honorable mention #4 (henceforth participant 6)

*Modeling the chikungunya epidemic in the Americas: Distributional ecology and population dynamics*

**Fig. 7:**
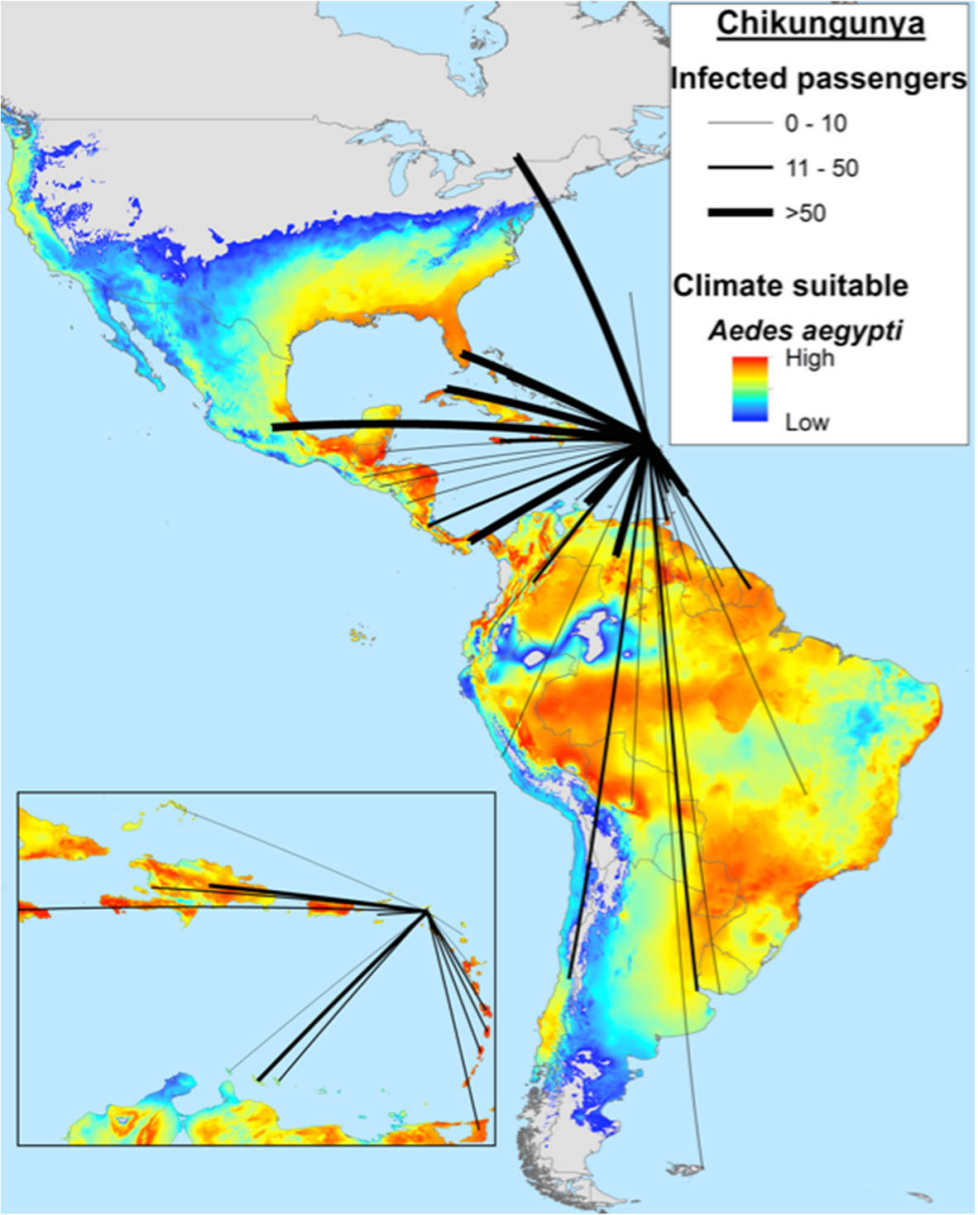
Identification of transmission hot spots as areas close to the niche centroid, showing human movement vectors (air travel) to estimate connectivity from Participant 6

*Modeling Approach*: Participant 6 used vector occurrence and climate variables [35] to generate ecological niche models (ENM) for vectors as multidimensional ellipsoid forms enclosing occurrences in a multidimensional environmental space, as described previously [36, 37]. The models depended on two main estimations: (i) rates at with which the virus is transmitted locally, and (ii) rates of importation of infections. To obtain these estimates, four “ingredients” were employed: primary occurrence data for mosquito species, 50-year climate data averages, estimated pairwise city-to-city airline passenger travel rates, and case report data from PAHO [30]. *Aedes aegypti* and *Aedes albopictus* occurrences were drawn from Campbell et al. [38]. Principal components analysis (PCA) was applied to the original climate variables to reduce their number and correlation [35]; the first three components (which explained 84.9% of the overall variance) were used as axes to define the multidimensional environmental space (NicheA 3.0 [39]). To identify areas with environmental conditions ideal for transmission [40–44], Participant 6 divided the ellipsoid for each vector into 100 layers summarizing proximity to the niche centroid to identify areas close to or far from the ENM centroid. Thus, areas close to the niche centroid (i.e., areas ideal for transmission) were identified as potential transmission hotspots (Fig. 7).

*Results*: Participant 6 found that most countries showed a dramatic pattern of intensive reporting in early weeks of the epidemic, followed by reduced reporting in later stages. This phenomenon was termed “surveillance fatigue” to refer to the reduction of collection, reporting, and publication of epidemiological data after explosive and sustained disease outbreak events. These models support the idea of higher incidences than those reported during late surveillance, suggesting that reduced reported rates may be driven by reduction in effort rather than a dramatic pause on local transmission. Countries closest to the centroid of vectors’ niches showed higher CHIKV prevalence. Fore a complete description of the model and methodology please refer to [45].

## Results

### Reported PAHO data

**Fig. 8:**
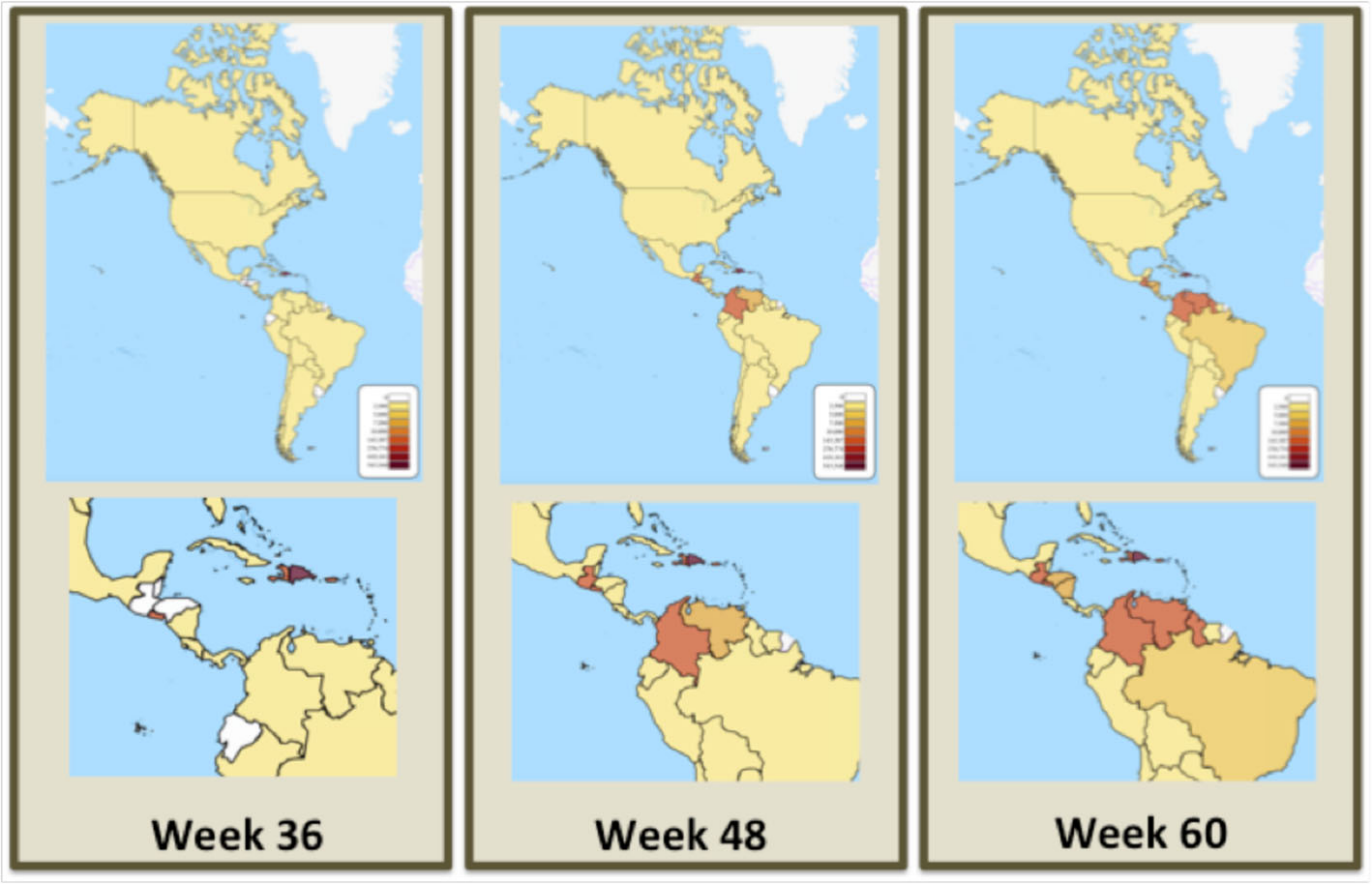
Progression of the CHIKV epidemic in the Americas as a function of time, as reported by participating countries to PAHO

The distribution of chikungunya cases across the 50 participating PAHO countries, at three times during the Challenge is shown in Fig. 8, to complement the weekly incidences shown in Fig. 1. An interactive version of this map, showing the CHIKV epidemic progression across the Western Hemisphere is available at the website: http://bsvgateway.org/chikv/ (courtesy and copyright, LANL). PAHO groups countries based on their geographic location into the following regions: North America (Bermuda, Canada, Mexico, USA); Central America (Costa Rica, El Salvador, Guatemala, Honduras, Nicaragua and Panama); Latin Caribbean (Cuba, Dominican Republic, French Guinea, Guadaloupe, Haiti, Martinique, Puerto Rico, Saint Barthelemy and Saint Martin (French Part); Andean Area (Bolivia, Colombia, Ecuador, Peru and Venezuela); South Zone (Argentina, Brazil, Chile, Paraguay and Uruguay) and the Non-Latin Caribbean countries (Anguilla, Antigua and Barbuda, Aruba, Bahamas, Cayman Islands, Curacao, Dominica, Grenada, Guyana, Jamaica, Montserrat, Saint Kitts and Nevis Saint Lucia, Saint Vincent and Grenadines, Saint Martin (Dutch part), Suriname, Trinidad and Tobago, Turks and Caicos, US Virgin Islands and UK Virgin Islands).

By week 36 of 2014 (corresponding to the week of September 6, 2014), at the beginning of the Challenge, 651,344 suspected cases were reported to PAHO, mostly in the Latin Caribbean region, with 8210 confirmed cases. The United States reported 762 imported cases. By week 48 of 2014, the epidemic was largely over in the Latin Caribbean region, but was peaking in Central America and the Andean region, with the total number of suspected at 914,960 and 15,906 confirmed cases. By the end of the Challenge in week 8 of 2015 (corresponding to the week of February 22, 2015), 1,247,359 cases had been reported to PAHO, of which 24,982 cases were confirmed. The epidemic had largely ended in the Latin Caribbean with a reported incidence of 2.2%, had subsided for the year in Central America with a reported incidence of 0.4%, and was still near a broad peak in the Andean area, with a reported incidence of 0.16%.

The 20 most-affected countries accounted for 98% of all reported chikungunya cases. The Dominican Republic reported the most cases, followed by El Salvador and Colombia. Both delayed and sporadic reporting were evident in the reported data, which should be kept in mind when this information is used to derive predictions of future epidemics. Accuracy and timeliness of the reported number of new cases may depend on the socio-economic structure, health care infrastructure, economic strength, and other factors.

We focused our discussion on a subset of the 50 PAHO countries with more complete data that allowed us to cross-check with alternative reports. The countries chosen represent the spectrum of variability associated with geography, socio-economic strata, population, weather and other parameters. Specifically, we analyzed Guadeloupe, Martinique, Dominican Republic, Haiti, United States, Mexico, El Salvador, Guatemala, Colombia, and Venezuela. Below, we present an analysis of solver entries for these countries. We chose to highlight different solver entries, including some that did not rank among the top 6, in the analysis presented in the manuscript. The reason being that certain submissions were more suitable for demonstration of a particular concept, and certain methodologies required attention, even though the entries did not rank among the top 6 solvers.

### Choice of models

To better understand the participant submissions, it is important to define and describe the general modeling approaches used by top participants. Classification of participant-submitted models was challenging, as participants typically used hybrid models that combined aspects of different approaches. For the purpose of this manuscript, and ensuing discussion, we have categorized the models submitted by all participants (not just the winning ones) into three broad categories: morphological models, mechanistic models, and subject matter expert models (SME). Morphological models represent a curve-fitting approach, wherein the curves can be defined analytically or via a set of differential equations. The curves are fitted independently to each outbreak and/or derived from an entirely different outbreak (e.g., dengue), suitably scaled and translated (solvers 1-4 in this manuscript). Mechanistic models attempt to capture the dynamic interplay of outbreaks in multiple countries and/or describe a dynamic interplay in the host (humans) and vectors (mosquitoes)(solvers 5 and 6 in this manuscript). The SME-based model (i.e., participant defined subject matter experts), utilized by only one participant (who did not rank in the top 6, not discussed in this manuscript), required consensus subjective opinion of various experts in the field, and did not require any type of computation to generate a prediction. This approach relied exclusively on expert judgment as an alternate to explicit modeling, leveraging the collective expertise to maximize forecast accuracy and simultaneously minimizing the number and strength of assumptions made. It is worth noting that this approach has been traditionally used by public health practitioners in the absence of models to inform their decisions. As expected from their descriptions, the model types overlap with each other in many cases. For example, many participants used subject matter expertise to inform mechanistic and morphological models.

### Data sources for effective predictions of Chikungunya

**Fig. 9:**
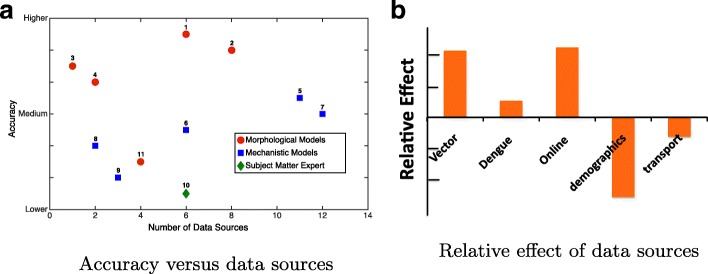
Effect of choice of data streams on accuracy of predictions, and relevant data streams for effective predictions. Participants 1-4 used morphological models, whereas 5 and 6 developed mechanistic models. **a** The effect of the number of data sources on accuracy of prediction, differentiated by the main categories of models employed by the participants. Note that the subject matter expert category refers to participant-defined subject matter experts. **b** The positive versus negative correlation of use of a data source on the accuracy of the prediction by regression analysis

Participants typically used several data sources to complement the information provided by PAHO. It is important to note that not all of these data sources were utilized to derive the predictions made in the final submissions. These data types included online web searches (e.g., Wikipedia, Google searches, government websites), climate information (e.g., temperature and humidity), vector-specific information (e.g., reporting of other mosquito-borne illnesses such as dengue in the same population, mosquito dynamics, ecology) and others (Table 2). Figure 9a represents the effect of the number of data sources used on the accuracy of prediction, as differentiated by the main categories of models defined elsewhere, for the top 10 participants of the Challenge. Participants with higher accuracy (i.e., 3, 4, 1, and 2) used anywhere between 1-8 data sources. However, not all data sources were considered or included in deriving the final prediction. Interestingly, all four of these top ranking participants used a morphological approach to arrive at their prediction.

**Table 2:** Major categories of data sources used by the top 6 participants in the DARPA Chikungunya Challenge, although not all data sources were incorporated into the modeling by the solvers

Solver #	PAHO	Online/	Population	Climate	Transportation	Economic	Vector	Dengue
		News				Index		
1	⋆			⋆	⋆	⋆		⋆
2	⋆	⋆⋆⋆		⋆⋆⋆		⋆		
3	⋆							
4	⋆		⋆					
5	⋆		⋆⋆	⋆⋆⋆	⋆		⋆⋆	⋆
6	⋆		⋆	⋆	⋆		⋆⋆	

There is no significant correlation between the number of data sources used and the accuracy of the forecasts, irrespective of the type of the model being utilized. In short, more data does not necessarily translate into better forecasts. The most important thing was to get the right kind of data, and to use the data appropriately. A regression analysis relating forecast accuracy to the types of data sources used by each participant (Figure 9b) showed that some data streams, such as those related to dengue epidemiology or mosquito dynamics, are used in models that have smaller forecasting errors. Conversely, models that exploit demographics and transportation data, have worse forecast accuracy than models that do not use them. Online searches correlated positively with accurate outcomes, although the specificity of this data-stream is difficult to define because of the wide variety of information types that can be tapped through the Internet. Arguably, the explanation is that Internet searches are used to validate, and sometimes, correct other data streams. In summary, not all data sources lead to improved forecasting accuracy. However, models that leverage specific data sources to substantiate missing links in surveillance data (e.g., dengue epidemiology data) or help improve data quality (e.g., Internet searches), typically have more accurate forecasts.

### Predicting the peak of the epidemic

**Fig. 10:**
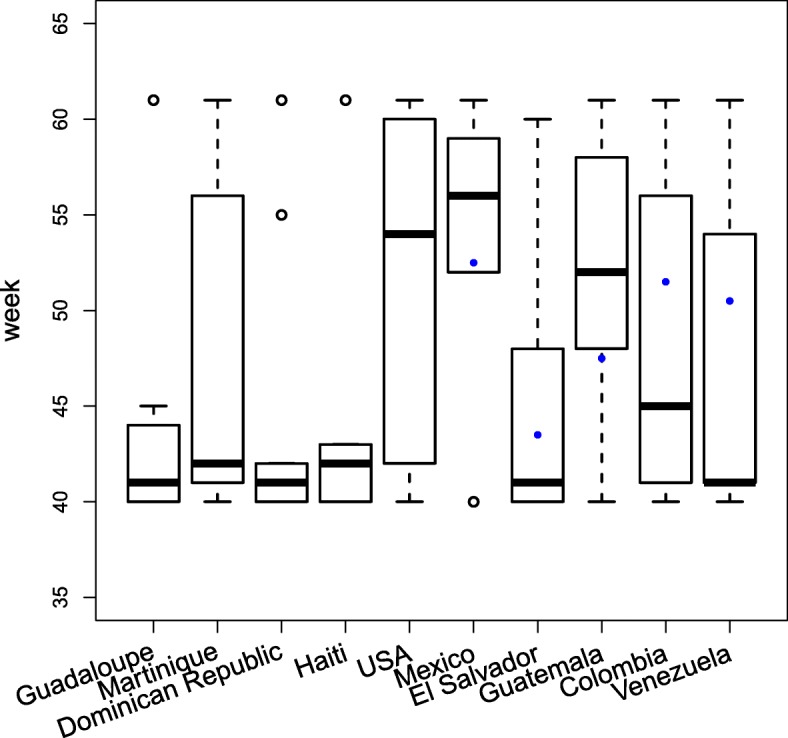
Peak Week Predictions. This figure shows the boxplot of peak week predictions for the top 9 participants for 10 countries. The box contains 50% of the predictions. The blue dots show the actual peak week as reported by PAHO (not shown for the first four countries because the epidemic had already peaked prior to week 35)

Although the peak of an outbreak is one of the most significant features of an epidemic, it was relatively difficult for the solvers to predict. We analyzed the peak predictions provided by the top 11 participants for the 20 hardest-hit countries. As mentioned earlier, by the time the first prediction was submitted, the epidemic had ended in the Latin Caribbean countries, and was just getting started in Central America and the Andean region. Since participants were not allowed to “back-cast” (i.e., predict in the past), the best choice was to select week 40 as the peak week, as a consequence of the challenge design. Figure 10 shows the peak predictions for a subset of countries. Only some of all 36 participants were able to accurately predict the exact week of the peak, and only in a few countries. The peak week as reported by PAHO clearly varies from participant submissions. A statistical analysis of the predicted peaks indicates that some participants showed very little variation (i.e., predictions were extremely conservative, and showed very little variability) in the predictions provided for all countries considered here (e.g., participants 1 and 4), whereas others showed more variation (e.g., participant 3) (data not shown). Indeed, the standard deviation for the PAHO data was larger due to the fact that the peak for these countries was spread out starting from week 8.5 for Saint Barthelemy to week 55 for Guyana (data not shown).

## Discussion

The ability to go beyond health surveillance and provide timely predictions of disease spread to mitigate disease outbreaks is a capability gap in global health. The DARPA Chikungunya Challenge (also referred to as the Challenge) attempted to address this gap by promoting innovation in data collection techniques and infectious disease modeling and prediction. The Challenge also aimed to identify and characterize methodologies, data streams, and approaches beyond the traditional winners that demonstrate critical value or lack thereof in predicting CHIKV outbreaks, with the intention of developing an integral multi-aspect forecasting system for future use.

It is a health security imperative to detect, contain, and prevent impacts of intentional or natural biological events. In order to accomplish this, proactive anticipation of the trajectory of infectious diseases outbreaks is required for public health planning. The results from this Challenge may inform future efforts in response to Zika outbreaks, or that associated with existing vector-borne diseases like dengue.

Although most participants utilized multiple data streams, the use of a large number of data streams did not necessarily improve the accuracy of the predictions. It was the choice of the data streams, and how they were utilized that enabled successful predictions. Participants that used alternative data streams to understand gaps and limitations in the available data were better able to predict the epidemic. Mosquito-dynamics, population specific information, and dengue-specific information correlated best with prediction accuracy.

## Conclusion

The results of this Challenge highlighted the fact that with careful consideration and understanding of the relative advantages and disadvantages of particular methods, implementation of an effective prediction system is feasible. Indeed, the ability of a model to forecast the reported data may not always translate into the ability of a model to forecast the epidemic. Furthermore, it may be of critical importance to also capture emergent behavior and mitigation strategies implemented in response to a deadly epidemic, which may require the use of more complex modeling approaches.

Improved data reporting might not always be possible, as this depends on the socio-economic and cultural framework of participating countries. However, uniform application of case definitions, reporting of geographic and demographic subsets of people, and reporting of dates of disease onset, rather than date of report may improve the overall usability of the reported data. Also, qualification of data with parallel epidemics (e.g., dengue, in this case) that rely on the same climactic factors and vector dynamics can significantly improve predictions. It is important for predictions to be judged against reliable reported data, such as a controlled test-bed, wherein the evaluation of different models and methodologies can be performed accurately and the value of various strategies clearly delineated. These findings, and further efforts to understand reported data and integrate multiple surveillance systems, could improve both the quality and quality of reporting and the associated response to an outbreak, making the dream of an effective infectious disease forecasting architecture a reality.
